# Guidelines for assessment of affect-related constructs

**DOI:** 10.3389/fpsyg.2023.1253477

**Published:** 2023-11-02

**Authors:** David M. Williams, Ryan E. Rhodes

**Affiliations:** ^1^Department of Behavioral and Social Sciences, Brown University School of Public Health, Providence, RI, United States; ^2^School of Exercise Science, Physical and Health Education, University of Victoria, Victoria, BC, Canada

**Keywords:** affective response, anticipated affect, affective associations, affective judgments, health behavior, assessment

## Abstract

Research on affect-related constructs as determinants of health behavior is increasing. The Affect and Health Behavior Framework (AHBF) provides a schematic structure to label, organize, and integrate affect-related constructs. To further facilitate research and theory development in health behavior science, the purpose of the present paper is to provide a critical review and guidelines for assessment of the affect-related constructs in the AHBF. The paper is organized based on the categories of constructs in the AHBF: Affective response to health behavior, incidental affect, affect processing, and affectively charged motivation. Future research should work toward parsing constructs where possible as well as identifying overlap. Researchers are advised to consider conceptual underpinnings and methodological nuances when assessing affect-related constructs in order to build a cumulative science of affective determinants of health behavior.

## Introduction

Historically, many theories focused on predicting, explaining, or intervening on behavior include a heavy emphasis on rationalist approaches (e.g., perceived benefits, perceived barriers, problem solving) in the social cognitive tradition (Conner and Norman, 2015). In health research, considerable emphasis in this approach can be traced to the health belief model (Rosenstock, 1974; Becker et al., 1975), with extensions to popular theories such as theory of reasoned action (Fishbein and Ajzen, 1975) and planned behavior (Ajzen, 1991) or social cognitive theory (Bandura, 1986). For example, in physical activity, social cognitive approaches represented over 50% of the theories applied in all behavioral prediction and intervention research up to 2010 (Rhodes and Nasuti, 2011).

However, in health behavior research, there has been a burgeoning interest in the role of dual process models that attempt to account for less rational and more impulsive or reflexive influences on behavior (Sheeran et al., 2013). While this includes a variety of different constructs (e.g., habit, identity, schema), affect and affect-related variables have been a major aspect of this rising research interest for both intervention (Rhodes et al., 2018; Williams et al., 2019) and prediction (Conner et al., 2015; Williams et al., 2018). Thus, the affect and health behavior literature has quickly become a crowded landscape of many variables (Hagger, 2014).

The Affect and Health Behavior Framework (AHBF) provides labeling, definitions, and organization of affect-related constructs that have been used in health behavior research, including affective response to a behavior, incidental affect experienced outside the context of the behavior, affective attitudes, anticipated affect, affective associations, implicit attitudes, and affectively charged motivational states, such as wanting and desire (Williams and Evans, 2014; Stevens et al., 2020). To further facilitate research and theory development in health behavior science, the purpose of the present paper is to provide a critical review (Grant and Booth, 2009) and guidelines for assessment of the constructs in the AHBF. While the literature is too vast to include a review of all measures of all affect-related constructs, our aim is to discuss fundamental considerations and highlight exemplar measures for each AHBF construct, as well as demonstrating differences and similarities among the constructs/measures.

## The affect and health behavior framework

**Figure 1:**
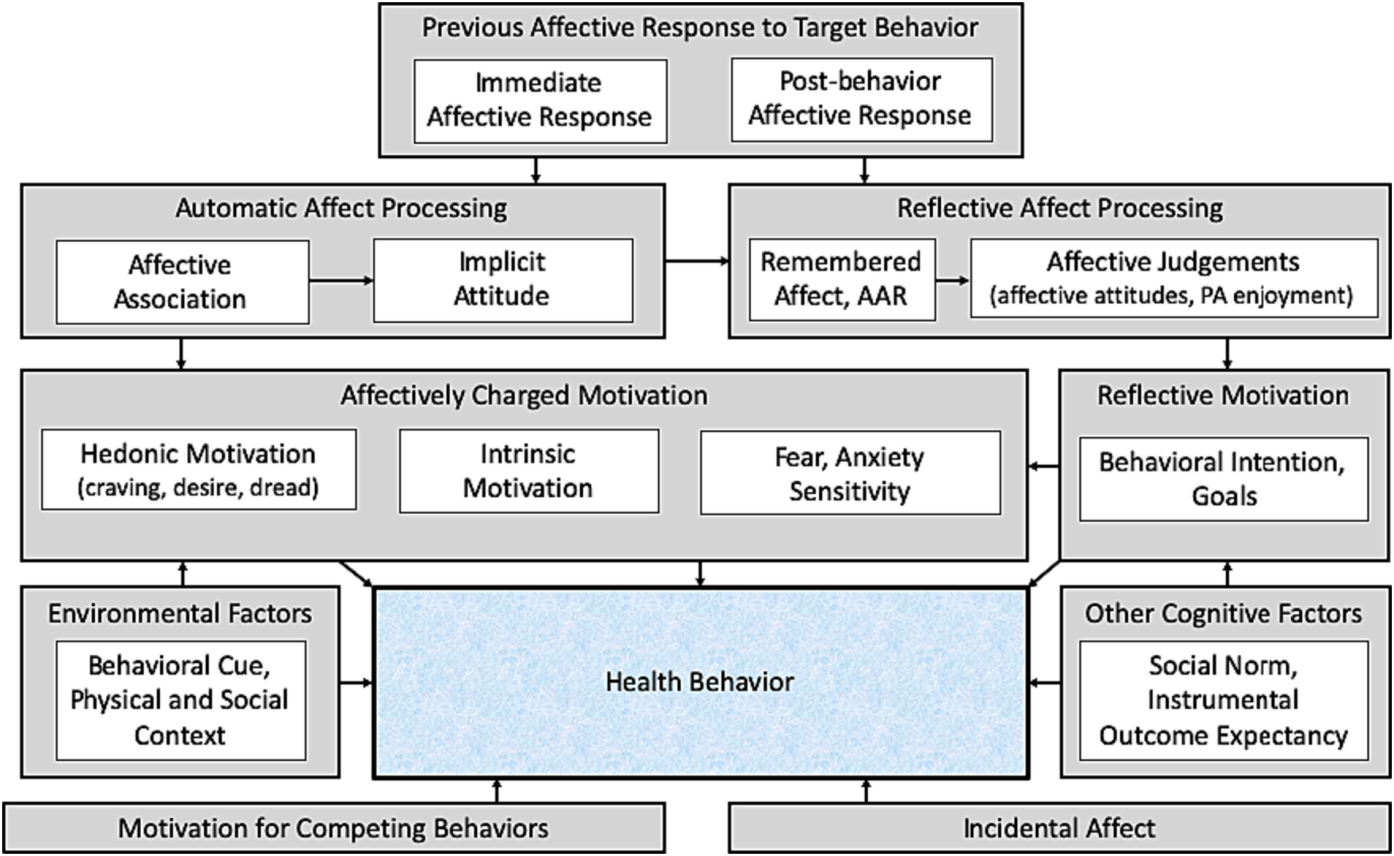
The affect and health behavior framework [AHBF, reproduced from Stevens et al., 2020; adapted from Williams and Evans, 2014]. AAR, anticipated affective response. PA, physical activity. Prior affective responses to a health behavior influence both automatic and reflective affect processing, which informs both affectively charged and reflective motivation to perform (or avoid) the behavior. Affect unrelated to the target behavior experienced throughout the day (incidental affect) also influences behavior, as do other cognitive factors, environmental factors, and motivation to perform other competing behaviors.

The AHBF (Figure 1) was created as a schematic structure to label, organize, and integrate the affect-related constructs used to understand health-related behavior (Williams and Evans, 2014). The term “affect-related constructs” is used because the AHBF includes affective responses to a target behavior, as well as the cognitive and motivational processes that do not constitute affect *per se*, but are necessary to translate affective responses into subsequent behavior. Specifically, according to the AHBF, a general causal pathway operates over time in which affective response to the target behavior (i.e., affective response) is translated into cognitive processing of affect (i.e., affect processing), and, in turn, motivation for subsequent performance of the target behavior (Williams and Evans, 2014). For example, people may feel immediate pleasure when indulging in chocolate cake or acute discomfort when engaging in a session of vigorous exercise. These affective responses to cake-eating and exercise determine cognitive associations and expectations that link the target behavior with prior experiences of the immediate affective response to the behavior. This affect processing then influences motivation for cake-eating or exercise the next time those behaviors are cued and available.

Each category of affect-related constructs—affective response, affect processing, and motivation—can be further divided. Specifically, affective response can be grouped into during-behavior and immediate post-behavior affective response. This distinction is important because affective valence often differs during versus immediately following a health behavior (Ekkekakis, 2013). Affect processing can be divided into automatic processing, such as affective associations and implicit attitudes, and reflective processing, such as anticipated affect and affective attitudes. The distinction between automatic and reflective affect processing is consistent with dual-processing theories (Evans, 2008; Hofmann et al., 2008) and accounts for differences between affect processing that is based on automatic non-conscious processing versus affect processing that is based on deliberate consideration of the affective consequences of behavior. Likewise, motivation can be divided into automatic or affectively charged motivation, such as craving, desire, or dread, versus reflective motivation, such as behavioral intentions or goals (Michie et al., 2011).

While some general causal pathways are proposed, the AHBF is an organizational framework rather than a theory *per se*. Therefore, it does not compete with other theories of affect, but can be used to facilitate research and further theorizing regarding the effects of affect-related variables on behavior. The AHBF is not rigid or static, but is open to modification as new constructs or connecting causal pathways are proposed, or as terminology or definitions are modified. Indeed, a few modifications to the original framework were made in a more recent version of the AHBF (Stevens et al., 2020). The term “affective judgments” was added as an umbrella term encompassing multiple reflective affective processing concepts, including affective attitudes and enjoyment (Rhodes et al., 2009). The categories of automatic and affectively charged motivation were merged, with hedonic motivation (Williams, 2018) added as an example of this category of motivational state. It should be noted that the specific affect-related constructs that are depicted in the schematic of the AHBF are not meant to be exhaustive but are examples of the various construct categories.

## Assessment of affect-related constructs

### Affective response to health behavior

The terms affect, core affect, mood, and emotion represent related but distinct concepts. *Affect* is an umbrella term encompassing evaluative psychophysiological states. All affective states have an underlying *core affect* component, characterized by a combined continuum of positive versus negative valence (i.e., pleasure versus displeasure) and high versus low arousal (Russell, 1980; Evmenenko and Teixeira, 2022). While awake, some level of core affect is always present, though often not in the forefront of consciousness. Additionally, core affect can be experienced as a component of more specific affective states, including *emotions* (e.g., anxiety, joy) and *moods* (e.g., sad, cheerful) that also involve cognitive appraisals and/or patterned physiological responses (Ekkekakis, 2013).

Consistent with the principle of psychological hedonism (Bentham, 1780/2007; Cabanac, 1992; Kahneman et al., 1997; Williams, 2018), research has consistently shown that a positive shift in affective valence (i.e., feeling more good/pleasure or less bad/displeasure) in response to a target behavior is predictive of increased likelihood of future performance of that behavior and a negative shift in affective valence (i.e., feeling less good/pleasure or more bad/displeasure) in response to a target behavior is predictive of decreased likelihood of future performance of that behavior. This is true across multiple health behavior domains, including, but not limited to eating (Stroebe et al., 2008), smoking relapse (Shiffman et al., 2006), continuation of exercise programs (Rhodes and Kates, 2015; Jones and Zenko, 2023), and greater alcohol use (Newlin and Renton, 2010).

There are numerous existing measures for assessing affective response. Affect measures can be categorized into those that assess specific affective states (i.e., moods or emotions), such as depression (Beck et al., 1961) or anxiety (Spielberger et al., 1970), and those that assess the full range of core affect, such as the Affect Grid (Russell et al., 1989), or a range of specific affective states intended to cover the full range of underlying core affect, such as the Positive Affect Negative Affect Schedule (PANAS; Watson et al., 1988). There are also behavior-specific measures for exercise, such as the Exercise-Induced Feeling Inventory (Gauvin and Rejeski, 1993) and the Physical Activity Affect Scale (Lox et al., 2000), and substance use, such as the Minnesota Tobacco Withdrawal Scale (Hughes and Hatsukami, 1986) and the Clinical Opiate Withdrawal Scale (Wesson and Ling, 2003). Here we highlight a few of the most important issues when selecting assessment content.

First, before deciding on assessment content, it is critical to decide on one’s conceptualization of affect, including whether one intends to assess core affect, or specific emotions or moods, as well as the underlying theory of affect, and the hypotheses under study (Ekkekakis, 2013). For example, if the goal of the research is to determine whether a more positive affective response to eating chocolate leads to more future chocolate consumption, then it would be appropriate to use the Feeling Scale (Hardy and Rejeski, 1989) to assess affective valence, as it is a single-item scale and therefore is easily administered during the behavior of interest. However, if the goal is to determine to what extent exercise alleviates depressive symptoms or smoking relieves anxiety, then it would be more appropriate to use a more targeted assessment of depression or anxiety.

Second, because affective response to health behavior, as well as incidental affect, must be assessed in real time, it is commonly assessed through ecological momentary assessment (EMA). In addition to real-time assessment, EMA allows for assessment of affect in research participants’ natural environments, thus minimizing biases inherent in assessments conducted in artificial environments (e.g., research laboratory) or contexts (e.g., with the researcher present). While EMA can be administered in a variety of ways, it is now almost always collected through mobile phone devices, which are conducive to real time assessments (i.e., during and immediately following the target behavior) in the respondent’s natural environments (i.e., not at the research laboratory), with ability to download and save data remotely. Additionally, EMA allows for collection of multiple instances of affective states across time and thus examination of patterns of affective response to a health behavior as well as incidental affect. Moreover, EMA is important for assessing affective response and incidental affect, as people are notoriously inaccurate in reporting how they felt at some specific point in the past (Kahneman et al., 1993). Indeed, retrospective recall of affective response to health behaviors, referred to in the AHBF as *remembered affect*, is an important determinant of behavior in its own right and is considered below in the section on *affect processing*. Thus, when assessing affective response, as opposed to remembered affect, it is important to obtain real-time assessments of affect (i.e., “how are you feeling now”) rather than affective recall (i.e., “how did you feel during the exercise you just completed”).

Third, examination of immediate and post-behavior affective response is not conducive to long questionnaires because it is infeasible and/or burdensome to administer multiple items during and immediately following a target behavior and at multiple random times throughout the day (i.e., incidental affect). There are, however, a few single-item measures that have been specifically designed and validated to assess acute and transient affect states, such as the Feeling Scale (Hardy and Rejeski, 1989) and Felt Arousal Scale (Svebak and Murgatroyd, 1985). Additionally, in many cases, researchers have selected one or a subset of items from previously validated affect scales to make the assessment more conducive to EMA and/or have modified the instructions from the original scales instructions so they are real-time instead of retrospective. Such modification of previously validated scales can be problematic, but also has trade-offs. Most affect questionnaires were created and validated without consideration of the constraints of EMA and therefore assess retrospective affective states over some period of time (e.g., the past week) and have multiple items that make them difficult to administer via EMA. In this case, the design of such questionnaires was undergirded (at least implicitly) by classical test theory, in which multiple items are necessary to extract a true score from extraneous measurement error (Spearman, 1904). However, when assessing real-time affective states, the assumptions of classical test theory may not apply if affective experience is viewed as an observable emergent property of affect rather than as an indicator of an unobservable latent affect construct (Coan, 2010). Nonetheless, use of such modified assessments should be validated as though they are new measures, without reliance on psychometric data from the previously validated scale.

Fourth, when assessing affective *response* to a behavior, it is important to assess the respondent’s baseline affect. Failure to do so may lead to mistaking individual differences in affective response to a behavior for individual differences in incidental affect—basal affective states that are unrelated to the target behavior. For example, a report of displeasure during exercise could reflect a response to the exercise stimulus or could simply be because the person generally has a low mood. When accounting for baseline affect, one approach is to assess the respondent’s affective state immediately prior to the target behavior. While this approach is simple and perhaps easiest, there is the possibility that affect leading up to the behavior will be impacted by one’s anticipation of performing the behavior. Indeed, though not included in prior versions of the AHBF, some researchers have referred to this as *anticipatory affect* (Knutson and Greer, 2008). To avoid the potential influence of anticipatory affect, and because incidental affect may change within and across days, it is best to sample the respondent’s incidental affect throughout the course of the day in order to understand the pattern of incidental affect leading up to performance of the target behavior. In addition to providing a baseline for affective response to the target behavior, incidental affect may have its own impact on the target behavior (Williams and Evans, 2014).

Finally, objective measures, such as facial electromyography, vocal acoustics, physiological changes (e.g., heart rate, cortisol, galvanic skin response), and brain activity (e.g., electroencephalography, functional magnetic resonance imaging, positron emission tomography) of affect and emotion (for a review see Quigley et al., 2014) can potentially be used to assess affective response to health behaviors. The advantage of such objective measures is that they are not open to the same biases as self-report measures of affect. However, if one theorizes that subjective experience is constitutive of affect, then objective measures, while potentially still minimizing reporting bias, cannot directly tap affective response (Cacioppo and Tassinary, 1990). Additionally, there are practical limitations to objective assessment of affect, including cost of equipment, need for specialized training, and feasibility of conducting such assessments outside the research laboratory in more ecologically valid environments.

### Affect processing

Affect processing is the cognitive processing through which previous affective responses influence motivation and, in turn, health-related behavior (Figure 1). While affective responses to health behaviors occur during and immediately following the target health behavior, affect processing—cognitive reflection or implicit processing of those affective experiences—can occur at any subsequent time point, and thus serves to causally connect previous affective responses to health behaviors to the motivation and ultimate decision to perform the target behavior the next time it is cued and available (Williams and Evans, 2014; Stevens et al., 2020).

The AHBF describes five main concepts underlying affect processing: affective associations, implicit attitudes, affective judgments, anticipated affective response, and remembered affect (Williams and Evans, 2014; Williams et al., 2018, 2019; Stevens et al., 2020). It is important to first highlight some overarching issues related to assessment of affect processing. First, whereas different types of affective response (i.e., immediate affective response, post-behavior affective response) differ only in respect to the timing of assessment and are thus all assessed in the same way, the five affect processing concepts are each different types of cognitive phenomena and thus are assessed in different ways.

Second, while affective responses are specific and transient affective states that occur in response to a target health behavior and thus are best assessed at the time that the affective state occurs, affect processing variables are reflective cognitive states and thus can be validly assessed at any time. For example, while someone’s affective response to smoking occurs and thus is best assessed at discrete points in time, their affective association (see next section) for smoking has no discrete temporal context and thus can be assessed at any time. Nonetheless, as with any psychological assessment, responses may be influenced by the context in which it is conducted, such as artificial laboratory environments or presence of researchers.

Third, while each affect processing variable is theoretically distinct from the others (as described below), there is the potential for common-method variance when more than one affect processing variable is assessed within a single study, as affect processing variables are typically assessed via questionnaires (with the exception of implicit attitudes) that ask respondents to report on affect-related phenomena.

#### Affective associations

Affective associations are associations that exist in memory between a behavior and previously experienced affective responses to that behavior (Kiviniemi and Klasko-Foster, 2018). While conceptually distinct from reflective affect processing constructs such as affective judgments, they are measured with self-report instruments so the two constructs may be difficult to separate in empirical tests (Sala et al., 2016). A key characteristic of affective associations is that negative and positive affective associations are separate constructs that can influence behavior and cognitions independently (Kiviniemi, 2018). This contrasts with prototypical measures of affective judgments, which include two evaluative poles within the same scaling. While there has been a limited volume of research using affective associations in health behavior, the current research has been applied to several different health behaviors including but not limited to physical activity (e.g., Murray et al., 2020), healthy eating (e.g., Walsh and Kiviniemi, 2014; Geers et al., 2017), health screening (e.g., Klasko-Foster et al., 2019), health self-examination (Brown-Kramer and Kiviniemi, 2015), smoking (e.g., Schutte and Marks, 2013), and condom use (e.g., Ellis et al., 2018).

Regardless of the health behavior under assessment, affective associations measurement begins with a prompt, such as “when I think about [behavior X], it makes me feel … ____.” This is followed by positive or negative affect words, rated on graded scales (e.g., 1 = not at all to 7 = extremely). The ratings for positive and negative affective association items are then aggregated to form a measure for each pole.

One of the key aspects of measuring affective associations, however, is to ensure the chosen words that are used in conjunction with the rating scales are good prototypes of the associated affective experience with a particular behavior. To accomplish this, some researchers have used prior elicitation research in the domain of interest. For example, in assessing affective associations for condom use, Ellis et al. (2018) used the words elicited from prior research on affective experiences (Crites et al., 1994). Other researchers have completed pilot studies to elicit key wording for affective associations. For example, Klasko-Foster et al. (2019) conducted a pilot study with a similar population to the main study to elicit words that correspond with affective associations of a colonoscopy exam. The words resulting from the pilot study were then used in the main study for ratings of positive and negative affective associations. Finally, some researchers have assessed affective associations with a hybrid approach (Schutte and Marks, 2013). In the technique developed by Peters and Slovic (1996) affective associations are measured by having respondents list key (with space for four answers) thoughts or images that came to mind when presented with the behavioral cue and then rate each thought/image on a scale (e.g., from 1 very negative to 5 very positive). This approach allows for idiosyncratic items for each respondent that are averaged to form an index of affective associations.

In summary, although there is a limited number of studies that have employed measures of affective associations, there is a wide range of health behaviors among these limited number of studies that have applied affective associations, and some initial support for the behavioral affective associations model (Kiviniemi and Klasko-Foster, 2018). The approach can be included in standard questionnaire packages, and typically include eight to 12 items to assess both positive and negative poles. The choice of affect-based words for respondents to rate is crucial for accurate assessment, and there are a variety of ways to ensure the items include appropriate words such as specific pilot research, prior validated items in the domain, and a hybrid elicitation and evaluation format.

#### Implicit affective attitudes

Implicit affective attitudes are the second of the two automatic affect processing constructs in the AHBF. An implicit attitude is the immediate affective evaluation of a behavior based on an accumulation of affective associations (Williams and Evans, 2014). Implicit attitudes are typically measured with reaction time tasks, and readers are encouraged to seek out overviews of various assessment methods (e.g., Gawronski and De Houwer, 2014; Gawronski and Hahn, 2018). As a cornerstone construct of dual process theories (Strack and Deutsch, 2004), implicit attitudes have been examined in numerous health behaviors such as physical activity (Chevance et al., 2019), alcohol use (e.g., Jones et al., 2018), healthy eating (e.g., Maas et al., 2015), and smoking (Dvorak et al., 2018; Cui et al., 2019). Below, we overview two implicit attitude measurement practices, evaluative priming and implicit association task (IAT), as examples of these approaches.

Evaluative priming is the oldest method of implicit attitude assessment (Fazio et al., 1986). In this assessment, the participant fixates at the center of a computer screen and places their index fingers over buttons they will press for future responses. The fixation point is then replaced with a priming of an affective stimulus, which can be expected as negative or positive in valence (e.g., junk food, vegetables, smoking, physical activity) or a neutral stimulus. The primes may be slow enough for conscious perception (e.g., > 200 ms) or subliminal perception (e.g., < 100 ms). Next, an affective stimulus that is either pleasant or unpleasant is presented for a longer period of time (e.g., 1,000 ms) and the individual must indicate the valence of the affective stimulus. Like affective associations noted above, reaction times are then averaged separately for pleasant and unpleasant stimuli. The expected measurement variance thus centres on the presumption that relatively faster reactions to pleasant stimuli indicate a positive affective implicit attitude toward the behavior and similar negative reactions represent a negative affective implicit attitude (Fazio et al., 1986).

The IAT is also a widely used test to assess implicit attitudes, with considerable recent research (Greenwald et al., 1998), now with several computer-based and online administrative options. Like evaluative priming, the test also assesses the strength of associations between two bipolar concepts. Specifically, bipolar targets (e.g., smoking-related vs. non-smoking-related) are paired with the bipolar attributes (e.g., pleasant vs. unpleasant), yielding two sets of dual evaluation categories (e.g., smoking/ unpleasant vs. non-smoking/pleasant and smoking/pleasant vs. non- smoking/unpleasant). Response time to categorize these targets is presumed to be shorter if the two concepts of the evaluation category (e.g., smoking/unpleasant) are strongly associated. This is often called the congruent condition. By contrast, the incongruent condition is when response time is longer between two concepts, and thus less paired association (e.g., smoking/pleasant). Differences in response time between the congruent and incongruent conditions is recognized as the implicit attitude (e.g., smoking cues relative to non-smoking cues).

For the IAT, target stimuli can involve a series of pictures or words typically presented in the center of the screen, along with paired words representing bipolar concepts (smoking vs. not smoking) and/or bipolar attributes (pleasant vs. unpleasant) on the upper left and right corners of the screen. Participants are typically instructed to quickly, and as accurately as possible, categorize the target stimuli using left or right response buttons with a fast interval separating responses (e.g., 150-ms). IAT protocol will generally include several practice trials for familiarization, followed by the actual test. The order of the congruent and incongruent conditions is typically counterbalanced (e.g., across participants and/or across sessions for each respondent), and keys responding are also sometimes interchanged between blocks of trials. Further, target stimuli are often quasi-randomized so that the pictures and words are alternated. The test generally takes between 6 to 12 min. When computing the difference score between congruent and incongruent response time trials, many researchers use the D score metric suggested by Greenwald et al. (2003) by computing the difference scores, and dividing it by the pooled standard deviations.

Like affective associations, implicit attitude tests rely on appropriate affective stimuli in which to provoke a response. Picture and word stimuli can be developed through pilot tests before the main study (e.g., Cui et al., 2019), or from previously developed and validated assessments and tested images.

Variants of the evaluative priming and IAT tests are also common. For example, the go-no-go association task (GNAT; Nosek and Banaji, 2001) is derived from the same logic as the IAT. The GNAT differs from the IAT in that it measures evaluations of a single category without necessitating a contrasting category. In typical GNAT trials, a series of stimuli are presented for brief durations (e.g., 600 ms), and when a stimulus belongs to either target category (which is displayed at the top of the screen), a response is required. No response is required if the stimulus belongs to neither category (i.e., a distractor). Similarly, the brief implicit association test (Sriram and Greenwald, 2009) measures associations between attributes and targets, like the IAT, but combines these within the same sets of trials to shorten the procedure. Variants all of these reaction time tests are too numerous to include here, suffice to say that many different variants of these types of assessments are proliferating in psychology (Gawronski and Hahn, 2018).

In summary, implicit affective attitudes, corresponding with the tenets of dual process theories, have been assessed among a wide range of health behaviors with evidence that these measures can show small, yet inconsistent associations with behavior, particularly when behavioral assessment is direct/objective (e.g., Hofmann et al., 2008; Chevance et al., 2019; Gallucci et al., 2020). Assessment involves various reaction-time tests which can be administered through in-person and online computer software, can be included in standard questionnaire packages, and typically take less than 15 min to administer. However, considerable debate continues on exactly what these reaction time measures actually assess and various limitations of these tests (Haines and Sumner, 2013; Conroy and Berry, 2017; Gawronski and Hahn, 2018; Znanewitz et al., 2018; Zenko and Ekkekakis, 2019; Schimmack, 2021). These criticisms include that the construct validity of the assessment is not fully developed, there is low reliability estimates in many cases, studies show low correlations among variants of these tests, and there are frequent interactions with contextual factors that show reaction-time tests are also dependent on many external (and thus not implicit) factors. Critical readers should consider these limitations when deciding upon the use of these reaction-time tests.

#### Affective judgments

Affective judgments is an umbrella term developed by Rhodes et al. (2009) to collectively include any construct that measures past reflections or attitudes regarding the pleasure, enjoyment, or fun associated with a target behavior. The construct is identical to experiential attitude (Fishbein and Ajzen, 2010), enjoyment (Kendzierski and DeCarlo, 1991) and affective attitude (Zanna and Rempel, 1988), and conceptually similar to satisfaction (Baldwin and Sala, 2018); all constructs of which reside in extended social cognition models (Rothman et al., 2009; Fishbein and Ajzen, 2010; Conner and Norman, 2015; Rhodes, 2021). Affective judgments are also commonly positioned as antecedents of intention; specifically, if one expects a health behavior will be pleasant and enjoyable, one is more likely to intend to act upon that behavior (Fishbein and Ajzen, 2010). Still, direct effects of affective judgments on health behaviors is also considered theoretically tenable when affective judgments serve as a proxy construct for more spontaneous hedonic-driven behavior, independent of preconceived intentional behavior (Rhodes and de Bruijn, 2013; McEachan et al., 2016; Rhodes and Gray, 2018). Affective judgments have been assessed as predictors and mechanisms of action in health behavior and health behavior change for over 30 years, including but not limited to physical activity, sedentary behavior, healthy eating, using condoms, sunscreen use, safe driving, drinking alcohol, smoking, using drugs, speeding, general health screening, breast self-examination, checking blood glucose levels, quitting smoking, breastfeeding, and blood donation (McEachan et al., 2016).

The most common measurement practice for affective judgments follows multi-item attitude assessment in the form of semantic differential bipolar or unipolar scaling (Fishbein and Ajzen, 1974; Ajzen, 2006). For example, bipolar affective judgment assessments include asking whether performing behavior X will be extremely unenjoyable to extremely enjoyable on a seven or five point scale (Conner and Norman, 2015). Alternatively, a unipolar scale may ask whether performing behavior X is enjoyable, with Likert (1932) response scaling from strongly disagree to strongly agree (Rhodes et al., 2010). The range and type of scaling have minimal differences on the predictive validity and internal consistency (Courneya et al., 2006; Rhodes et al., 2006, 2010). Best practice, however, includes details within each item regarding the behavioral target, action, context, and time elements (Fishbein and Ajzen, 2010). Affective judgments typically include affective wording to rate the expected behavioral experience such as pleasant (unpleasant), enjoyable (unenjoyable), fun (boring), stimulating (unstimulating), among other synonyms; typically the construct can be measured reliably with three to five of these summed descriptors (Rhodes et al., 2009; Nasuti and Rhodes, 2013).

Affective judgments can also be measured with specific enjoyment scales. For example, Kendzierski and DeCarlo (1991) developed the 18-item physical activity enjoyment scale (PACES) to specifically measure affective judgments relating to exercise and physical activity. Items were generated from literature reviews of enjoyment, dictionary entries, and qualitative inquiry with several samples on enjoyment experiences in physical activity. The instrument has been used in considerable research and is highly correlated with the measures of affective attitude noted above (Rhodes et al., 2009). Like many affective attitude measures, the PACES asks respondents to consider their current behavioral practices by rating bipolar items on a seven point scale [e.g., (1) I enjoy it to (7) I hate it; (1) I find it pleasurable to (7) I find it unpleasurable; (1) it’s a lot of fun to (7) it’s no fun at all, etc.].

In summary, affective judgments correspond with social cognitive representations of affective experiences and thus reside in well-researched social cognitive models (e.g., as affective attitude or enjoyment). There is considerable literature validating their simple measurement structure compared to the other constructs of the AHBF. Furthermore, their predictive efficacy with health behaviors and intentions is now well-established (McEachan et al., 2016), yet the independent contribution of affective judgments on health behavior with other constructs of the AHBF is an area of considerable current research inquiry (Baumeister et al., 2007; Conner et al., 2015; Rhodes and Kates, 2015; Hamilton et al., 2020). Prototypical measurement of affective judgments includes semantic differential attitude assessment, yet specific enjoyment measures are common. Like affective associations, the choice of affect-based words for respondents to rate is crucial for accurate assessment, and there have been many studies using factor analyses to distinguish the more instrumental attitude descriptors from those which are affective (Zanna and Rempel, 1988; Crites et al., 1994).

#### Anticipated affective response

Anticipated affective response is the expectation of how one will feel in response to engaging in, or failing to engage in, a health behavior. It is conceptually similar to anticipated regret (Simonson, 1992) or anticipated affective reaction (Conner et al., 2015), and is a type of outcome expectation (Fishbein et al., 2001). Conner et al. (2015) propose that the theoretical process for how anticipated regret affects health behaviors occurs via self-conscious emotions (e.g., guilt; Giner-Sorolla, 2001), although Rhodes and Mistry (2016) have demonstrated that some regret stems from simple anticipation of missed opportunities and not personal shame. Regardless of its possible antecedents, research has shown that post-behavior feeling states that are anticipated pre-emptively can serve as motivation to avoid that outcome (Simonson, 1992; Brewer et al., 2016), and have influence independent of affective judgments (Conner et al., 2015). The concept of anticipated affective response has been applied to numerous health behaviors, including vaccination, cancer screening, physical activity, condom use/safe sex, speeding/unsafe driving, smoking, healthy eating, alcohol/drug abuse, and sunscreen use, among others (Brewer et al., 2016).

Measurement of anticipated affective response has used questionnaire items. For example, Conner et al. (study 2; 2015) applied a single item to assess 20 different health behaviors in terms of anticipated affective response: “I will feel regret if I do NOT engage in X over the next four weeks” scored on a seven point scale, from “definitely no” to “definitely yes.” As another example, Ferrer et al. (2015) applied two anticipated affective response questions to predict receiving threatening genetic risk information about disease. These included “If I found out that my genes put me at high risk for a fatal disease, I would be devastated” and “I do not think I would be able to cope with finding out that my genes put me at high risk for a fatal disease,” scored on seven-point Likert-type scales.

A more elaborate investigation of anticipated affective response includes both potential positive and negative affective experiences anticipated from a health behavior. For example, Conner et al. (2013) measured anticipated negative affective responses to blood donation with three items (e.g., “If during the next six months I did not give blood again, I would regret it, very unlikely–very likely”) and positive anticipated affective responses (e.g., “If I were to give blood again during the next six months, I would be proud, very unlikely–very likely”). These items were scored between 1 and 5 and summed for a total scale value (Conner et al., 2013).

Variant measures have also included modified assessments of self-conscious emotions scales. For example, Gilchrist and Sabiston (2018) measured anticipated affective response to successful intention translations of physical activity with the modified eight-item authentic and hubristic pride–fitness subscales of the body- related self-conscious emotions–fitness instrument (Castonguay et al., 2016). The researchers modified the stem from experienced pride to anticipated pride.

In summary, anticipated affective responses correspond with social cognitive representations of expectancy and thus align with processed affect in social cognitive models. There is support for the construct validity of anticipated affective responses when compared to affective judgments in health behavior research (Conner et al., 2015). Furthermore, there is also considerable literature validating their predictive efficacy with health behaviors and intentions (Brewer et al., 2016). Prototypical measurement of anticipated affective responses includes questionnaire items asking about either negative (e.g., regret, shame, guilt) or positive (e.g., pride) anticipation of self-conscious emotions. While many variants of measurement have shown reliability and predictive validity, studies that used multi-item measures with regret as the key affective phrasing, may be superior in predictive validity of health behavior to single-item measures or those that used other self-conscious emotions in the phrasing of the items (Brewer et al., 2016).

#### Remembered affect

Remembered affect is the specific recall of an affective response to a behavior (Kahneman et al., 1993). The concept differs from an actual affective response because it is remembered (i.e., cognitively processed) and not an *in situ* experience, and differs from affective judgments and anticipated affective response because it is a specific memory of an experience and not an aggregate appraisal of affective experiences with a behavior. The concept has been tested less than all other affect processing variables in the AHBF, but it holds conceptual interest because it allows for explorations of singular affect processing experiences on health behaviors.

Remembered affect is typically assessed by adapting core affect measures (see prior section on affect response assessments) to ask about a prior behavioral experience. For example, Cox et al. (2020) assessed remembered affect with an adapted feeling scale measure (Hardy and Rejeski, 1989), of the level of pleasantness or unpleasantness that participants remembered experiencing during a recent exercise session. Specifically, on a scale ranging from −100 (most unpleasant imaginable) to 100 (most pleasant imaginable), participants answered the question “How did the exercise session in the laboratory make you feel?” Higher scores indicate more positive remembered affect. Similarly Kwan et al. (2017) applied an adapted physical activity affect scale (Lox et al., 2000) and feeling scale to assess remembered affect. Participants were asked “Think back to how you felt while exercising today. How do you remember feeling while exercising today?” and then responded using the feeling scale and physical activity affect scale measures.

In summary, remembered affect is the recalled affective response to a specific behavioral experience, which may hold utility in understanding very specific anticipated affective responses. Research using this approach in health behavior is limited at present and typically explored in lab-based protocols with the stimulus behavior. While conceptually distinct from other AHBF constructs, more research is needed to understand whether the construct is distinct from generalized anticipated affective responses, affective judgments, and *in situ* affective responses.

### Affectively-charged motivation

Consistent with dual-processing theory (e.g., Strack and Deutsch, 2004; Evans, 2008; Hofmann et al., 2009), the AHBF distinguishes between affectively charged and reflective motivation types (Williams and Evans, 2014; Stevens et al., 2020; Williams, 2023). Affectively charged motivations have also been referred to as automatic motivations and defined as “affectively charged desires to perform or dread of performing a behavior that is associated with previous immediate pleasure (or reduced displeasure) or immediate displeasure (or reduced pleasure), respectively,” whereas reflective motivations are “affectively cold motivations to perform or not perform a behavior based on conscious and deliberate consideration of the outcomes of the behavior” (Williams, 2023) (see also Michie et al., 2011; Hofmann and Nordgren, 2015).

Affectively charged motivations span multiple literatures across health psychology, including the concepts of automatic wanting from incentive salience theory (Berridge and Robinson, 2016), craving and urge from theories of addiction (Sayette et al., 2000; Sayette and Wilson, 2015), intrinsic motivation from self-determination theory (Ryan and Deci, 2000), as well as more recent conceptualizations of desire (Hofmann and Van Dillen, 2012; Hofmann and Nordgren, 2015), and hedonic motivation (Williams, 2018).

Traditionally, affectively charged motivations have been studied in the context of behaviors that bring immediate pleasure or alleviate displeasure, but are risky or unhealthy, such as addictive behaviors (Robinson and Berridge, 1993; Koob and Le Moal, 1997; Kavanagh et al., 2005; Hofmann and Van Dillen, 2012). However, while less often studied, affectively charged motivations are also likely relevant to health-promoting behaviors such as healthy eating, regular physical activity, and avoiding addictive substances and risky sexual behavior (Fishbein, 1979; Rogers, 1983; Bandura, 1986; Ajzen, 1991).

Of key importance for assessment of affectively charged motivation is its dissociation from reflective motivation (Williams, 2023). Importantly, affectively charged motivation, while typically (though perhaps not always; see Berridge and Robinson, 1995) consciously experienced, is not (or at least need not be; see Tiffany and Conklin, 2000) a function of conscious and deliberate processing. For example, one may have an affectively charged or automatic desire for the chocolate cake, while at the same time holding a deliberately formulated intention to avoid the cake and instead eat the healthier fruit salad.

Affectively charged motivation is also conceptually distinct (though perhaps not as easily empirically distinguished, see below) from the concepts of affective response and affective judgements. The distinction between affectively charged motivation and affective response is undergirded by distinct neurobiological underpinnings (Berridge, 2004) as well as distinct psychological experiences (Williams, 2018). The pleasure of biting into a piece of chocolate cake and the discomfort of vigorous exercise are different experiences than the hedonic desire to eat the chocolate cake and the hedonic dread of exercising. Besides the fact that, in these examples, pleasure or displeasure occurs *while* eating or running, and desire or dread occurs *before* the behavior, the experiences of hedonic response and hedonic motivation *feel* different. While the pleasure of eating chocolate cake may lead to future hedonic desire for chocolate cake, the experience of pleasure has no necessary motivational quality in its own right. Conversely, hedonic motivation has no necessary affective quality to it. Hedonic motivation may coincide with a feeling of pleasure, particularly when the object of hedonic motivation is within reach; or with displeasure, when the object of hedonic motivation is likely to go unfulfilled. But these affective qualities (pleasure or displeasure) that *may co-occur with* hedonic motivation are not *defining qualities* of the experience of hedonic motivation. For example, the smell of a chocolate cake baking may trigger hedonic motivation for the chocolate cake as a function of *previous* pleasure from eating chocolate cake. However, one may or may not experience pleasure in the present, at the time they experience hedonic motivation for the chocolate cake.

Affectively charged motivation also differs from affect processing in that the latter are cognitive reflections on or expectations of the affect experienced in response to a target behavior, whereas the former represent automatic desires to perform the behavior. The three categories of constructs (affectively charged motivation, affect processing, and affective response), though distinct, are nonetheless related in the process of reward in which previous affective responses to a stimulus become associated with said stimulus through affect processing, resulting in future affectively charged motivations when the stimulus is cued (Figure 1).

#### Craving and urge

Under the affectively charged motivation umbrella is the concept of craving from addiction research (Sayette et al., 2000; Drummond, 2001; Skinner and Aubin, 2010; Sayette and Wilson, 2015; Sayette, 2016). Some version of automatic associative processing of reward contingencies is inherent to many prominent theories of craving (Solomon and Corbit, 1974; Wikler, 1980; Siegel, 1983; Drummond et al., 1990; Robinson and Berridge, 1993; Koob and Le Moal, 1997; Redish, 2004; West, 2007). In the context of craving theories, positive affective responses include both increased pleasure and reduced displeasure—the latter often a function of relief from withdrawal symptoms—in response to a drug or food that becomes associated with environmental (e.g., alcohol advertising) or psychological (e.g., depressed mood) cues that subsequently trigger craving. Importantly, this associative process happens automatically, without the need for deliberate consideration of the immediate pleasure (or relief from displeasure) that may result from consumption of the target substance.

Craving is typically assessed via one-item or brief multi-item self-report measures, which are often administered in real-time (i.e., how much are you craving a cigarette right now?) using oral, written, or computer-administered questionnaires in the context of laboratory research or in field-based research using EMA (Shiffman, 2009; Serre et al., 2015). The advent of EMA methods allows for frequent real-time—rather than retrospective—assessments capturing within-day fluctuations in craving, including changes that occur temporally proximal to opportunities to engage in the target behavior.

#### Incentive salience or ‘wanting’

Incentive salience theory is a theory of motivation that emphasizes the distinction between automatic ‘wanting’ and ‘liking’, which represent the neurobiological processes that underly automatic motivation and affective response, respectively (Berridge and Robinson, 2016). Consistent with the emphasis on neurobiological processes, much of the research inspired by incentive salience theory employs neuroscience methods among non-human animals. This research has supported the theorized distinction between ‘liking’ (affective response) and ‘wanting’ (automatic motivation) and the critical role of ‘wanting’ as a determinant of behavior, particularly eating and addictive behavior (Berridge, 2004).

In addition to neurobiological research among non-human animals, incentive salience theory has inspired neuroimaging and experimental laboratory research among humans in which ‘wanting’ (i.e., incentive salience) is often assessed with behavioral measures, such as amount of or willingness to work for the behavioral outcome that is the target of hedonic motivation (Pool et al., 2016). While indispensable in research that attempts to dissociate ‘liking’ and ‘wanting’, these measures are not as useful for research in which behavior is the dependent variable because they confound hedonic motivation with behavior. Alternatively, ‘wanting’ has been assessed by simply asking people to rate on numerical scales “How much do you want to eat this item right now?” (Born et al., 2011) “How much do you want to eat it?” (Bushman et al., 2011) Or, “I want to eat the food very much” versus “I do not want to eat the food at all” (Jiang et al., 2008). These measures provide a simple self-report method for assessing automatic motivation. However, a weakness of this approach is that responses may tap either automatic or reflective motivation, or some combination of the two.

#### Desire

Hofmann and colleagues’ (Hofmann et al., 2008; Hofmann and Van Dillen, 2012) have adapted the reflective-impulsive dual-processing framework (Strack and Deutsch, 2004) to the context of health behavior, in which “Desire can be defined as an affectively charged motivation toward a certain object, person, or activity that is associated with pleasure or relief from displeasure” (Hofmann and Van Dillen, 2012, p. 317) (see also, Kavanagh et al., 2005). Though the authors’ original framework (Hofmann et al., 2008) did not emphasize the distinction between desire and more cognitively oriented forms of motivation, Hofmann and Nordgren (2015) later contrasted the concept of desire with cognitively derived goals—a position that is consistent with the distinction between automatic and reflective motivation in the AHBF.

Hofmann et al. (2012) and Bagozzi et al. (2003) have assessed self-reported desire in field-based research using ecological momentary assessment and traditional questionnaires, respectively. From an assessment standpoint, relative to the word “want,” colloquial use of the word “desire” may more easily distinguish automatic motivation from reflective motivation. That is, colloquially, one is more likely to say that she *desires* a beer, chocolate cake, a cigarette, or sex with an attractive acquaintance (i.e., typically hedonic desires), than to say that she *desires* to be able to go to work in the morning, to stick to her diet, to quit smoking, or to remain faithful to her partner (i.e., typically reflective desires).

However, while Perugini and Bagozzi (2004) showed that a questionnaire assessment of “desire” was empirically distinguishable from assessment of “intention” (see also Perugini and Conner, 2000), other research has shown that the two constructs are highly correlated (Rhodes et al., 2006). Thus, while perhaps less ambiguous than self-reported wanting, assessments of self-reported desire may still be conflated with intentions, goals, and other aspects of reflective motivation, as indicated by previous conceptual parsing of desire subtypes (Davis, 1982; Schueler, 1995; Schroeder, 2004).

#### Hedonic motivation

The concepts of ‘wanting’ and ‘dread’ from incentive salience theory represent the neurobiological underpinnings of *hedonic motivation*—the felt *hedonic desire* to produce an immediate behavioral outcome that has previously brought immediate pleasure (or relief from displeasure) or a felt *hedonic dread* of producing an immediate behavioral outcome that has previously brought immediate displeasure (or reduced pleasure). Hedonic motivation encompasses the neurobiological concept of ‘wanting’, but also emphasizes the psychological experience of hedonic motivation as a manifestation of its neurobiological underpinnings (Panksepp and Watt, 2011). In the theory of hedonic motivation, hedonic motivation is positioned as the proximal mechanism through which *previous* hedonic responses influence future behavior (Williams, 2018; Williams et al., 2019).

A self-report measure(s) of the experiential aspect of hedonic motivation is needed that is distinct from reflective motivation. Such a measure could be used in research on the psychological experience and self-report of hedonic desire (and dread) to mirror research on the psychological experience and self-report of pleasure (and displeasure). The development of a self-report measure of hedonic motivation will be difficult though because of the apparent absence of colloquial terms that distinguish between hedonic and reflective motivation.

In two ongoing studies, the present authors are testing the validity of a hedonic-reflective motivation scale intended to assess and parse hedonic and reflective motivation for exercise (Connell Bohlen et al., 2023; Williams et al., in press). The first part of the scale includes four items beginning with the root: “On days when I plan to exercise I am motivated to exercise…” followed by one hedonic motivation item (“without thinking about the reasons for it”) and three reflective motivation items (“because I know it’s good for me,” “because I made a commitment to it,” and “because I will feel bad if I do not”). The second part of the questionnaire asks respondents to rate two additional hedonic motivation items: (a) “I would want to exercise regularly even if there were no benefits to exercising” from “not at all” to “definitely” and (b) “On days when I plan to exercise I usually…” “completely dread exercising” to “completely look forward to exercising” with a neutral point of “neither dread nor look forward to exercising.” We are also assessing hedonic and reflective motivation via EMA with two (“How much are you looking forward to exercise today?”; “How much are you dreading exercise today?”) and one item (“How much do you feel like you should exercise today?”), respectively (Bohlen et al., 2023).

#### Additional measures of affectively charged motivation

Finally, two additional measures that do not fit into any of the above subcategories, appears to tap the broader concept of affectively charged motivation. The attraction-antipathy scale from the Affective Exercise Experiences Questionnaire (Ekkekakis et al., 2021) asks respondents to rate five items (e.g., “exercise is a tempting activity” versus “exercise is an uninviting activity”) on 7-point bipolar response scale with a middle neutral point. Likewise, the 13-item Cravings for Rest and Volitional Energy Expenditure (i.e., CRAVE) scale asks respondents to rate on an 11-point scale ranging from “not at all” to “more than ever” how much they “want or desire” to engage in physical activity (e.g., “move my body,” “be physically active”) and sedentary behavior (e.g., “do nothing active,” “just sit down”) (Stults-Kolehmainen et al., 2021).

## Recommendations and future directions

Table 1 provides a guide for assessment of affect-related constructs. As with selection of measures in other areas of study, deciding among which measures to use is a matter of research question(s), theoretical or conceptual model, research methods, and practical constraints of the study design. The first step is deciding what affect-related construct(s) one wants to assess (e.g., immediate and post-behavior affective response, affective associations). This should be determined by one’s research question(s) and theoretical or conceptual model. The AHBF (Figure 1) suggests causal pathways through which affect-related constructs are interrelate and connect with other constructs to influence health behavior. For example, one may propose that affective associations for aerobic exercise mediates the effects of immediate affective response to exercise on future exercise behavior.

**Table 1:** Information for assessing constructs from the Affect and Health Behavior Framework.

	Theoretical underpinnings	Example measures	Method
Affective response			
During−/post-behavior	Psychological hedonism	Feeling scale, felt arousal scale, affect grid	Ecological momentary assessment (EMA)
Incidental affect	Broaden and build, Affect-regulation theory, Affect congruency	Feeling scale, felt arousal scale, affect grid	EMA
Affect processing			
Affective associations	Behavioral affective associations model	Positive or negative affect words, rated on graded scales; affective exercise experiences core affective experiences subscale	Paper or electronic questionnaire
Implicit affective attitudes	Dual-processing theory	Evaluative priming, Implicit association test, Go-no-go association task	Computer task
Affective judgments	Social cognitive theories	Semantic differential, enjoyment measures	Paper or electronic questionnaire
Anticipated affective response	Social cognitive theories	Anticipated regret, personal shame, positive and negative affective experiences anticipated from a health behavior	Paper or electronic questionnaire
Remembered affect	Peak-and-end rule of remembered affect	Adapted feeling scale, felt arousal scale, affect grid to a single remembered experience	Paper or electronic questionnaire
Affectively-charged motivation			
Craving	Addiction theories	One-item measures of craving; food craving questionnaire; alcohol craving questionnaire; questionnaire on smoking urges	EMA; Paper or electronic questionnaire
Incentive salience or “wanting”	Incentive salience theory	One-item measures of “wanting”; behavioral measures of willingness to work; neurobiological assessments of dopamine release in non-human animals	EMA
Desire	Elaborated intrusion theory	One-item measures of desire	EMA
Intrinsic motivation	Self-determination theory	Behavioral regulations questionnaire, intrinsic motivation questionnaire	Paper or electronic questionnaire
Hedonic motivation	Theory of hedonic motivation	Hedonic-reflective motivation measure; attraction-antipathy scale from the affective exercise experiences questionnaire	EMA; Paper or electronic questionnaire

The second step is to select an assessment strategy (e.g., timing of assessments) that adequately captures the construct(s) of interest given the research question(s). Following from the above example, one may choose to assess immediate affective response to exercise during a one-week training period, followed by assessment of affective associations at the end of the one-week period, and then assessment of free-living exercise behavior during the subsequent week. This would allow for a test of the hypothesized causal chain linking immediate affective response to exercise, affective associations, and exercise behavior. It is important to recognize that appropriate timing of assessment may differ depending on qualitative differences in the behavior. For example, though all forms of exercise, there are different temporal considerations for assessing affective response to resistance training (Andrade et al., 2022), aerobic interval training (Box et al., 2020), and stretching (Henriques et al., 2023).

The third step is to select the specific measures of affect-related constructs given the constraints of the study design. Continuing with the example, one may use the Feeling Scale to assess immediate affective response to exercise, delivered via app-based EMA if the exercise is being completed in participants’ natural environments. Affective associations could be assessed using the affective associations scale (Kiviniemi, 2018) administered via paper-pencil or on-line questionnaire.

The authors’ respective research teams are currently conducting research in which multiple affect-related constructs are assessed, thus allowing for examination of multiple causal pathways proposed in the AHBF. In a randomized trial examining the effects of self-paced versus moderate intensity exercise prescriptions on exercise behavior among midlife adults, Connell Bohlen et al. (2023) are using EMA to assess affective response to exercise, incidental affect, and hedonic motivation for exercise. In the context of the smart-phone app that delivers the EMA protocol, participants also respond to self-report assessments of affective judgments, anticipated affective response, remembered affect, and hedonic motivation. As another example, Rhodes and colleagues have explored the multivariate application of affective judgments and the affective response to a bout of exercise to predict post-partum mothers (Rhodes et al., 2023). The results showed that the constructs may predict behavior independently and differ over time.

In building a cumulative science of affective determinants of health behavior, it will be important to continue to differentiate between constructs from conceptual and methodological perspectives, while at the same time recognizing where there is overlap in conceptualization and ability to empirically distinguish among constructs. It also important to recognize that some research may be intended to maximize prediction of behavior, in which case parsing from among the many affect-related constructs is less important, whereas other research may emphasize distinction among affect-related constructs and thus the total variance explained in a behavior is less important. Indeed, for interventions to be effective, it is necessary to understand the full range of affect-related constructs, how they interrelate to influence behavior, and thus which are most likely targets in a given intervention context (Dunton et al., 2023).

Finally, greater consideration and/or discussion of the role of language in affect-related research is also warranted. As noted above, research is needed to distinguish between self-report measures of hedonic and reflective motivation. However, language is central to both individual comprehension and articulation of experience, and by extension, assessment of affect-related constructs depends on semantics. Likewise, cultural differences in health behaviors and their antecedents requires research that considers the influence of culture on the meaning of terms used to describe affect-related constructs and the implications for assessment.

## Author contributions

DW: Conceptualization, Writing – original draft, Writing – review & editing. RR: Conceptualization, Writing – original draft, Writing – review & editing.
